# Centrifugal Spinning Enables the Formation of Silver Microfibers with Nanostructures

**DOI:** 10.3390/nano12132145

**Published:** 2022-06-22

**Authors:** Xujing Zhang, Songsong Tang, Zhaokun Wu, Ye Chen, Zhen Li, Zongqian Wang, Jian Zhou

**Affiliations:** 1 Key Laboratory for Polymeric Composite & Functional Materials of Ministry of Education, Guangzhou Key Laboratory of Flexible Electronic Materials and Wearable Devices, State Key Laboratory for Optoelectronic Materials and Technologies, School of Material Science and Engineering, Sun Yat-sen University, Guangzhou 510275, China; xzhangxj@163.com (X.Z.); songsongtang@outlook.com (S.T.); yzwzk613@gmail.com (Z.W.); 2 School of Textiles and Garment, Anhui Polytechnic University, Wuhu 241000, China; 3 State Key Laboratory for Modification of Chemical Fibers and Polymer Materials, Donghua University, Shanghai 200051, China; chenye@dhu.edu.cn; 4 Foshan City Zhongrou Material Technology Co., Ltd., Foshan 528225, China; lizhenzuokeyan@163.com

**Keywords:** centrifugal spinning, silver nanowires, nanofibers, annealing

## Abstract

Silver nanowires (AgNWs) have received much attention and application in transparent electrodes, wearable electronic devices, and sensors. The hope is for these nanowires to eventually replace the most commonly used transparent electrode material—indium tin oxide (ITO). However, electrospinning used for the preparation of AgNWs on a large scale is limited by its low productivity and high electric field, while the alcohol-thermal method is limited to mixing by-product silver nanoparticles in silver nanowires. We demonstrate a novel and simple centrifugal spinning approach in order to successfully fabricate ultra-long silver microfibers based on AgNO_3_ and polyvinyl pyrrolidone (PVP). The centrifugal-spun precursor fiber and silver fiber can be prepared to as thin as 390 and 310 nm, respectively. Annealed fibers show typical nanostructures with grains down to a minimum size of 51 nm. Combinations of different parameters, including concentrations of PVP, needle size, and annealing temperature are also investigated, in order to optimize the spinning process of ultra-long silver microfibers. The feasibility of preparing silver microfibers by centrifugal spinning is preliminarily verified, examining prospects for mass production. Furthermore, numerous strategies related to assisting the creation of silver nanofibers using centrifugal spinning are presented as possibilities in future development.

## 1. Introduction

Nanofibers with diameters ranging from tens to hundreds of nanometers have piqued researchers’ interest, due to their excellent mechanical properties, high specific surface area, and so on [1,2,3,4]. Such fibers have found widespread applications in tissue engineering scaffolds, high-performance filtering materials, drug delivery, artificial blood vessels, biochips, nanosensors, composite materials, and in other emerging fields [1,5,6,7,8]. Although electrospinning may be used to create nanofibers with great precision, this method’s low productivity, specific equipment, and high electric field prevent it from being utilized on a broader scale. Meanwhile, melt-out, phase separation, template synthesis, and self-assembly are all rather complicated processes; they can only be employed to make nanofibers from a restricted number of materials. Thus, it is still challenging to prepare nanofibers on a large scale and in ultra-long lengths using a straightforward method.

Centrifugal spinning, inspired by marshmallow preparation technology, has been researched to manufacture microfibers, or even nanofibers [9]. Compared to the traditional method, centrifugal spinning has been recognized as a high-productivity (average output per nozzle is 60 g/h), low-cost fiber manufacturing process that balances the centrifugal force of the spinning solution with the characteristics of the fluid. The method does not require a very high electric field, and is environmentally friendly [1,10]. Although it has been widely investigated and focuses on preparing disordered nanofibers, there are still some unexplored areas; for example, preparing specific nanofibers (silver nanowires, metal nanowires, conductive nanofibers, etc.) with unique functions needs to be explored [10,11]. Among the nanofibers, metal nanowires have emerged as promising candidates for micromechanics, nanoelectronics, and optoelectronics, due to their exceptional densities of states and high aspect ratios. Notably, silver, with the highest electrical and thermal conductivity in 1D form (AgNWs), has shown promising potential for practical applications in various technologies, including wearable electronics, smart sensors, optoelectronic displays, energy harvesting devices, and catalysis [11,12,13,14,15,16]. Despite substantial research into the manufacture of AgNWs, producing ultra-long AgNWs with high yields continues to be a challenge, hampered by the restriction of hydrothermal, solvothermal, and template preparation methods. Although the alcohol-thermal approach provides reasonable control over the morphology of AgNWs, straightforwardly producing ultra-long nanowires with high yields remains a challenge.

Unlike the traditional method, centrifugal spinning can produce an ultra-long silver precursor fiber, which can then be transformed into a silver fiber after post-treatment without the requirement of high electric fields and environmental pollution. Here, we use centrifugal spinning to create ultra-long silver microfibers, and an annealing process with polyvinyl pyrrolidone (PVP) polymer that functions as a fiber polymer backbone, as a stabilizer of Ag^+^ in solution, and as a reducing agent in the annealing process. The formation mechanism of ultra-long silver fibers based on centrifugal spinning and annealing is elaborated. At the same time, the uniform distribution of Ag on the surface of fibers with ultra-long length illustrates that centrifugal spinning combined with an annealing process is an effective method for large-scale preparation of silver microfibers. Furthermore, the morphology of the nanostructures on the silver microfiber is further studied by varying the PVP loading, needle size, and annealing temperature. At the same time, strategies for decreasing centrifugal spun fiber, in addition to developmental trends for centrifugal spinning, are proposed.

## 2. Materials and Methods

### 2.1. Materials

Silver nitrate (AgNO_3_, 99.8%) was purchased from Guangzhou Chemical Reagent Factory (Guangzhou, China); polyvinyl pyrrolidone (PVP, Mw = 1300 K, K88–96) and acetonitrile (99.5%) were obtained from Alladin Ltd. (Shanghai, China). Sodium dodecyl sulfate (SDS, 98.5%) was supported by Biofroxx Co., Ltd. (Guangzhou, China). All reagents were used without further purification.

### 2.2. Preparation of Silver Nanowire Using Centrifugal Spinning and Annealing

**Figure 1:**
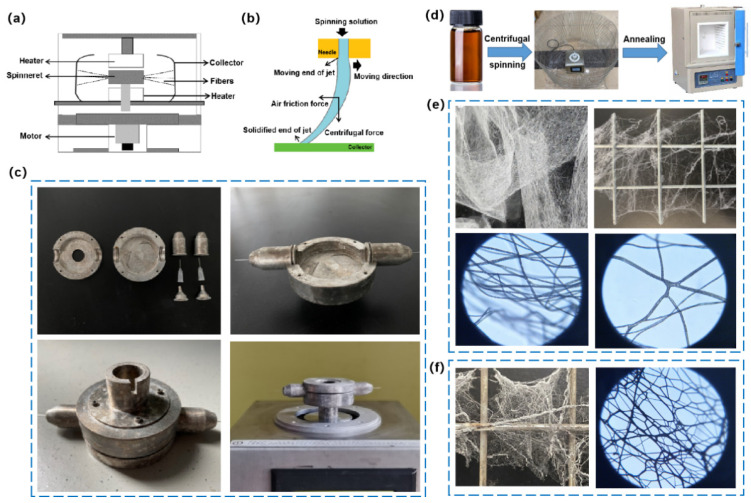
Centrifugal spinning principles, device, and procedure for preparing ultra-long silver microfibers. (**a**) A schematic diagram of the centrifugal spinning equipment. (**b**) Force diagram of a spinning fluid. (**c**) Homemade centrifugal spinning apparatus based on a spinning coater. (**d**) Schematic representation of silver microfibers produced by centrifugal spinning and post-annealing. (**e**) Photographs of centrifugal-spun silver precursor fibers. (**f**) Photographs of centrifugal-spun silver precursor fibers after annealing.

A specific amount of PVP was dissolved in 5 mL of acetonitrile and stirred vigorously for 2 h at room temperature. The solution was then vigorously stirred for 4 h at ambient temperature with 1 mL of distilled water, 3 g of AgNO_3_, and 0.1 g of SDS to produce a brown spinning solution, while the weight ratios between PVP and AgNO_3_ were set to 4:15, 5:15, and 6:15. The spinning solution was loaded into homemade centrifuge spinning equipment (Figure 1c) equipped with 30 G (0.16 mm), 32 G (0.11 mm), and 34 G (0.063 mm) needles, and spun. The rotation fluid reservoir was designed and manufactured to adapt to a SPIN 51 spin coater (Shanghai chemat advanced ceramics technology company, Shanghai, China). The distance between the collector (stainless steel mesh) and the spinning needle was set to 30 cm, while the spinning speed was set to 6000 rpm. After collecting the silver precursor fiber (Figure 1e) on the stainless mesh was placed into the muffle furnace, heated to the target temperature for 30 min, and held for 2 h before cooling down to ambient temperature in 60 min, while the annealing temperatures were set to 250, 280, 300, and 350 °C. Then, the silver microfiber was obtained (Figure 1f).

### 2.3. Characterization

Cold-field emission scanning electron microscopy (SU8010, Hitachi, Tokyo, Japan) was used to investigate the morphology of the aerogels. The long fiber with or without annealing was fractured into short pieces and attached to the SEM stage via carbon adhesive tape. Meanwhile, the average diameter of the fiber with or without annealing was estimated by ImageJ software at counts of more than 100 fibers. X-ray diffraction (XRD) was performed in a continuous mode using a PANalytical X-ray diffractometer (Malvern Panalytical, Malvern, UK) with Cu Kα radiation (λ = 1.5406 Å) at 40 kV and 15 mA. The diffraction patterns were acquired with a step length of 0.02° and a scanning speed of 4° min^−1^ over an angular range of 30–90°.

## 3. Results

### 3.1. Fibrillation of the Silver Nanowire

It is challenging to increase the nanowire length while using preparation procedures to manufacture silver nanowires with excellent shape. In order to obtain the ultra-long silver microfibers, we use centrifugal spinning (Figure 1) with an annealing process using the assistance of PVP, a copolymer with a reducibility function that aids in preparing ultra-long microfibers. Centrifugal spinning facilitates the preparation of ultra-long precursors of the silver microfibers by forming an ultra-long PVP/AgNO_3_ composite fiber; meanwhile, the PVP reduces Ag^+^ to Ag, based on its reducibility under the annealing process.

As a synthetic water/acetonitrile-soluble polymer with an ultra-high molecular weight (PMw = 1,300,000), PVP has the general properties of facilitating water-soluble polymer colloid protection, film formation, bonding, hygroscopicity, solubilization, or condensation. It serves as the fiber’s backbone and as an Ag^+^ stabilizer in the spinning solution. The spinning solution was first loaded into the rotating spinning container (Figure 1a–c) equipped with a spinning needle for centrifugal spinning. After that, the motor starts and drives the container to rotate. When the rotating speed reaches a critical value, centrifugal force overcomes the surface tension of the spinning fluid, ejecting a liquid jet from the spinning head’s nozzle tip. The jet is then stretched and eventually deposits onto the collector, resulting in solidified nanofibers [4]. Moreover, the PVP is commonly used to aid in the preparation of AgNW and nanoparticles because of its coordination of the Ag^+^ ion with the O atom of the PVP carbonyl group [17], which facilitates electron exchange between the Ag^+^ and the adjacent N atom on the pyrrolidone ring; meanwhile, the N atoms with lone pair electrons act as electron donors, eventually reducing Ag^+^ to form PVP-capped Ag [18]. Furthermore, during the alcohol-thermal synthesis process, the molarity and molecular weight (Mw) of PVP impact AgNW production [19]. In one PVP macromolecule, more carbonyl groups and silver ions are coordinated along the long chain of PVP. It is proposed that a higher molecular weight PVP should more easily induce the PVP-Ag coordination compound to arrange into a one-dimensional state.

In order to achieve the production of ultra-long silver microfibers, PVP was first dissolved in 5 mL of acetonitrile by vigorous stirring for 2 h at room temperature, as shown in Figure 1d,g. The homogeneous solution was then mixed with 1 mL of distilled water, 3 g of AgNO_3_, and 0.1 g of SDS, and vigorously stirred for 4 h at room temperature to produce a brown spinning solution, which was loaded into a homemade centrifuge spinning apparatus equipped with a 32 G needle. The distance between the collector (stainless steel mesh) and the spinning needle was 30 cm, while the spinning speed was 6000 rpm. Fibers with average and minimum diameters of 1.88 and 0.51 µm, respectively, were collected. The collected fiber on the stainless steel mesh was then placed into the muffle furnace, heated to 250 °C for 30 min, and held for 2 h before cooling to ambient temperature for 60 min, yielding ultra-long silver nanofibers and microfibers which have an average and minimum diameter of 1.43 and 0.31 µm, respectively.

### 3.2. Effect of PVP on the Morphology of the Silver Nanofiber

**Figure 2:**
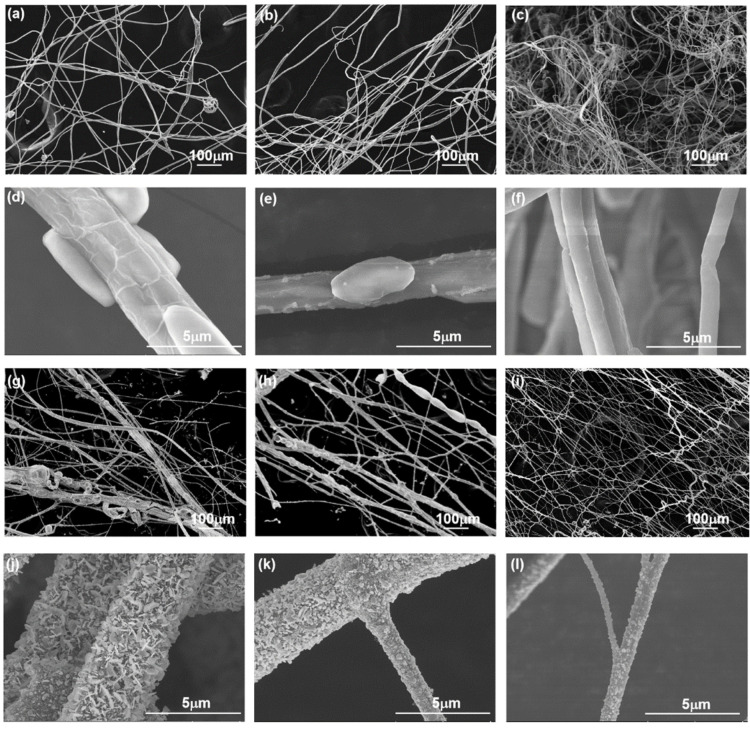
Morphology of the centrifugal-spun silver precursor fibers and annealed silver fibers. SEM images of centrifugal-spun silver precursor fibers with PVP:AgNO_3_ weight ratios of 4:15 (**a**), 5:15 (**b**), and 6:15 (**c**). High magnification of SEM images related to centrifugal-spun silver precursor fibers with PVP:AgNO_3_ weight ratios of 4:15 (**d**), 5:15 (**e**), and 6:15 (**f**). SEM images of annealed silver fibers with PVP:AgNO_3_ weight ratios of 4:15 (**g**), 5:15 (**h**), and 6:15 (**i**). High magnification of SEM images related to annealed silver fiber with PVP:AgNO_3_ weight ratios of 4:15 (**j**), 5:15 (**k**), and 6:15 (**l**).

**Figure 3:**
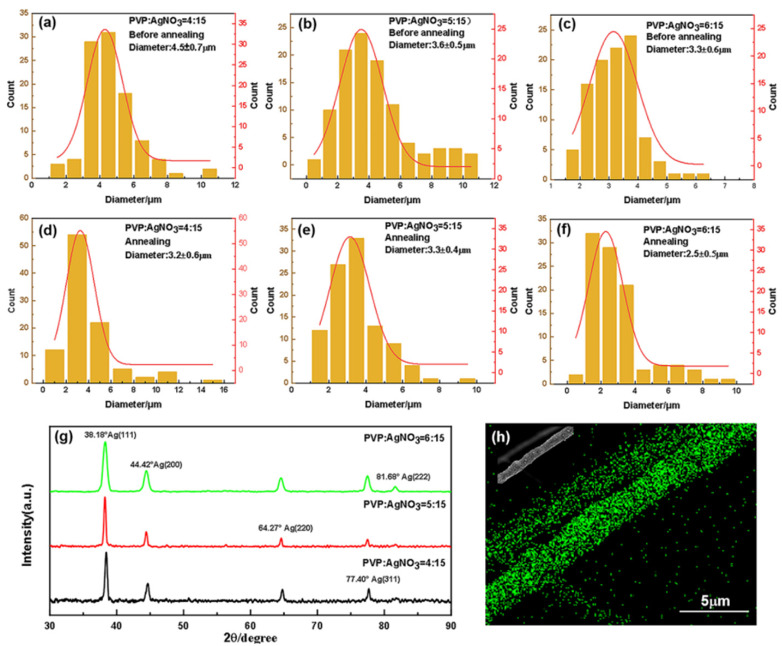
Diameter analysis and physical characterization of centrifugally spun microfibers made with different PVP/AgNO_3_ weight ratios. Diameter of centrifugal spun silver precursor fibers at a weight ratio of PVP:AgNO_3_ at 4:15 (**a**), 5:15 (**b**), and 6:15 (**c**). Diameter of annealed silver fibers at a weight ratio of PVP:AgNO_3_ at 4:15 (**d**), 5:15 (**e**), and 6:15 (**f**). (**g**) The XRD patterns of annealed silver fibers were prepared from different PVP/AgNO_3_ weight ratios. (**h**) The EDS image of annealed fibers shows the Ag element distributed on the fiber.

SEM images of the silver precursor fibers are shown in Figure 2a–f and Figure S1a–c. The diameter distribution of the silver precursor fiber that was generated by centrifugal spinning (Figure 3a–c) shows significant dispersion, indicating a multi-scale structure. The diameter of the silver precursor fibers was 3–5 µm on average, and the minimum diameter of the fiber was 0.39 µm. Noticeably, the average diameter of silver precursor fibers reduces as PVP loading increases. The spinning continuity varies significantly, although fibers can be spun from spinning solutions with varying PVP:AgNO_3_ ratios (4:15, 5:15, and 6:15). The weight ratio of PVP:AgNO_3_ at 4:15 give the worst spinnability of spinning solution, with shorter fibers of various diameters being collected. Conversely, when the weight ratio of PVP:AgNO_3_ in the spinning solution is 6:15, the spinnability is optimal, the fiber length is longer, and the fiber diameter is relatively uniform. The average length of the resulting fibers increases as the solute concentration of the spinning solution increases due to the increased co-stretching effect of the air resistance-spinning head pulling on the fibers, which results in smaller fiber diameters.

Figure 2g–i and Figure S1d–f show the SEM image of the annealed silver fibers. It can be seen that the average diameter (Figure 3d–f) of the obtained annealed silver fiber is between 3–4 µm, whereas the annealed silver fiber that was prepared under a PVP:AgNO_3_ ratio of 6:15 has a minimum diameter (0.63 µm). Noticeably, the overall diameter of the fibers decreases after annealing because of the decomposition of PVP and SDS under high temperatures, while the average diameter of annealed silver fiber decreases as PVP content increases. According to Figure 2 and Figure S1, when the weight fraction of PVP in the spinning solution is up to 6:15, the fiber has a relatively complete network structure and a relatively uniform thickness.

Figure 2d–f and Figure S2 show that the bead-string structure is suspended on the surface of the silver precursor fiber. This phenomenon is probably ascribed to the spinning jet’s extended flow converting the coiled macromolecules in the dissolved polymer into an oriented, entangled network that persists as the fiber solidifies, followed by a surface tension-driven shrinkage of the jet radius. The bead-string structure becomes less prominent as the PVP concentration increases. This is due to the higher viscosity of the spinning solution in conjunction with the decrease in surface tension. Furthermore, when comparing the SEM images of silver precursor fiber and annealed fiber prepared under various ratios of PVP:AgNO_3_, the annealed fiber has a rougher surface than the silver precursor fiber, which is a result of the Ag^+^ being reduced to Ag and attaching to the surface of annealed fibers during the annealing process. The morphology of the Ag particles on the different annealed fibers differed, with the Ag on the surface of annealed fiber under PVP:AgNO_3_ = 4:15 having a sheet-like shape with a large aspect ratio (length from 98 to 2230 nm). Meanwhile, the Ag on the surface of annealed fiber made with PVP:AgNO_3_ = 5:15 and PVP:AgNO_3_ = 6:15 had a compact spherical shape with a small aspect ratio. Moreover, the Ag particles on the surface of annealed fiber prepared under PVP:AgNO_3_ = 5:15 had a size of 70–2280 nm, which was larger than the Ag particles on the surface of annealed fiber prepared under PVP:AgNO_3_ = 6:15 (51–766 nm).

Figure 3g depicts the X-ray diffraction (XRD) patterns of annealed silver fibers under varied PVP loading. The crystalline planes of (111), (200), (220), and (311) correspond to four typical peaks at 38.35°, 44.42°, 64.27°, and 77.40°, respectively (JCPDS file No. 04-0783) [20,21,22,23]. As a result of the low PVP loading, the samples are cubic-crystal structure metallic Ag nanoparticles attached to the fiber’s surface, with no observable diffraction peaks of 81.68°, attributable to the crystalline plane of the fiber (222). As the PVP loading increases, the peak of 81.68° becomes higher and sharper, while no diffraction peaks of Ag_2_O are detected in this pattern; this indicates that PVP aids in the growth of Ag nanoparticles, and protects silver nanoparticles from oxidation [24]. Meanwhile, the sharp and strong peaks indicate that silver nanoparticles have high crystallinity, which implies that high purity silver nanoparticles are prepared through centrifugal spinning and annealing. Based on the XRD patterns, the crystal size of the Ag nanoparticle was calculated using D = Kλ/Bcosθ, where D is the average crystallite size; K is the Scherrer constant; B is the full width at half maximum intensity of the peak; θ is the diffraction angle; and λ is the X-ray wavelength (1.54056 Å). The size of the Ag nanoparticle prepared with PVP:AgNO_3_ = 5:15 was calculated to be 25.2 nm, which was larger than the size of the Ag nanoparticle prepared with PVP:AgNO_3_ = 4:15 (22.9 nm) and with PVP:AgNO_3_ = 6:15 (17.3 nm). The PVP aids in the preparation of Ag nanoparticles and protects them from oxidation; however, an excess of PVP loading reduces the size of the Ag nanoparticle crystal. Furthermore, the EDS image of the silver microfibers (Figure 3h and Figure S3) revealed that the silver nanoparticles are evenly distributed on the fibers.

### 3.3. Effect of Needle Size on the Morphology of the Silver Microfiber

**Figure 4:**
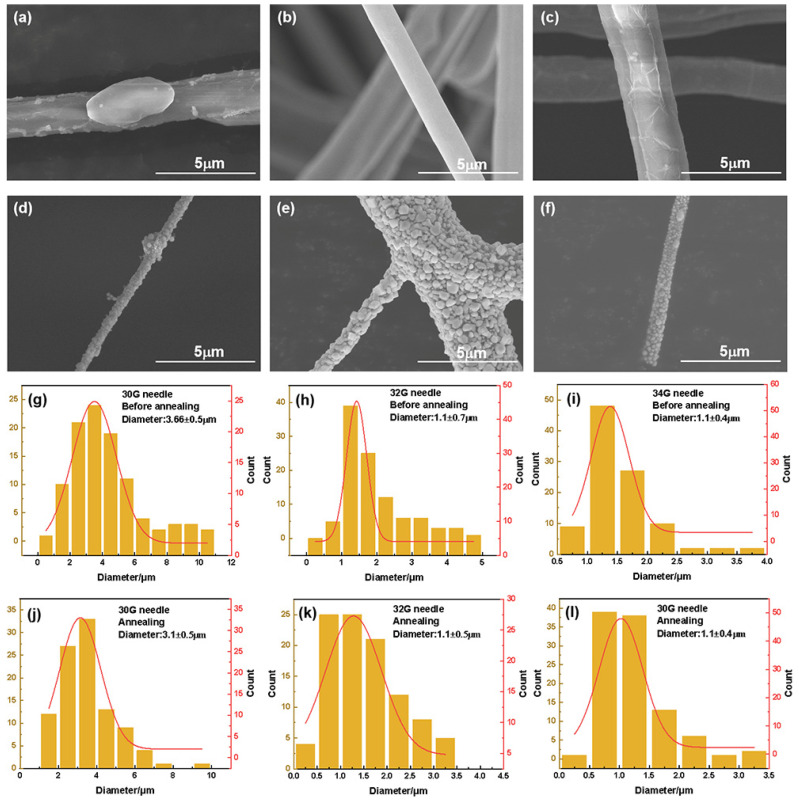
Characterization of centrifugally spun microfibers based on different needle sizes. SEM images of centrifugal-spun silver precursor fibers from needle sizes of 30 G (**a**), 32 G (**b**), and 34 G (**c**). SEM images of annealed silver fibers from needle sizes of 30 G (**d**), 32 G (**e**), and 34 G (**f**). Diameter distributions of centrifugal-spun silver precursor fibers from needle sizes of 30 G (**g**), 32 G (**h**), and 34 G (**i**). Diameter distributions of annealed silver fibers from needle sizes of 30 G (**j**), 32 G (**k**), and 34 G (**l**).

The distribution of fiber diameters obtained with different sizes of the needles is shown in Figure 4g–l. The average fiber diameter decreases with the needle diameter, while annealed silver fiber has a smaller diameter than silver precursor fiber. Annealed silver fibers prepared with 30 G, 32 G, and 34 G needles have diameters of 1–5, 0.4–3.5, and 0.3–1.2 µm. Figure 4a–f, Figures S2 and S4 show SEM images of the silver precursor fiber before and after annealing. The silver precursor fiber has high homogeneity before annealing, according to Figures S2 and S4, and the thinner needle manufactured silver fiber has a superior shape of the bead. Moreover, the 30 G and 32 G needles can be used for continuous spinning. Conversely, the needle becomes readily blocked when utilizing a 34 G needle, lowering production efficiency dramatically. Furthermore, a 34 G needle prepares the annealed silver fiber with an average diameter of 1.23 µm, which is smaller than that of fiber prepared by 30 G (4.15 µm) and 32 G (1.88 µm) needle, illustrates that different fiber sizes can be achieved by adjusting the needle size.

The size of the needle had an effect on the development of the Ag particle on the surface of the annealed fiber, similar to the effect observed with PVP loading. However, due to the adoption of PVP:AgNO_3_ = 5:15, the Ag on the surface of different annealed fibers generated under different needles showed particles forming. The silver branch (480–570 nm) and aggregation powder (1.04–1.25 μm) are present on the surface of annealed fiber manufactured with a 30 G needle. Meanwhile, the Ag particles are evenly distributed on the surface of fiber produced with 32 G and 34 G needles. The reason for this is because when the diameter of the fiber increases with the size of the needle, AgNO_3_ aggregation in the silver precursor fiber increases.

### 3.4. Effect of Annealing Temperature on the Morphology of the Silver Microfiber

**Figure 5:**
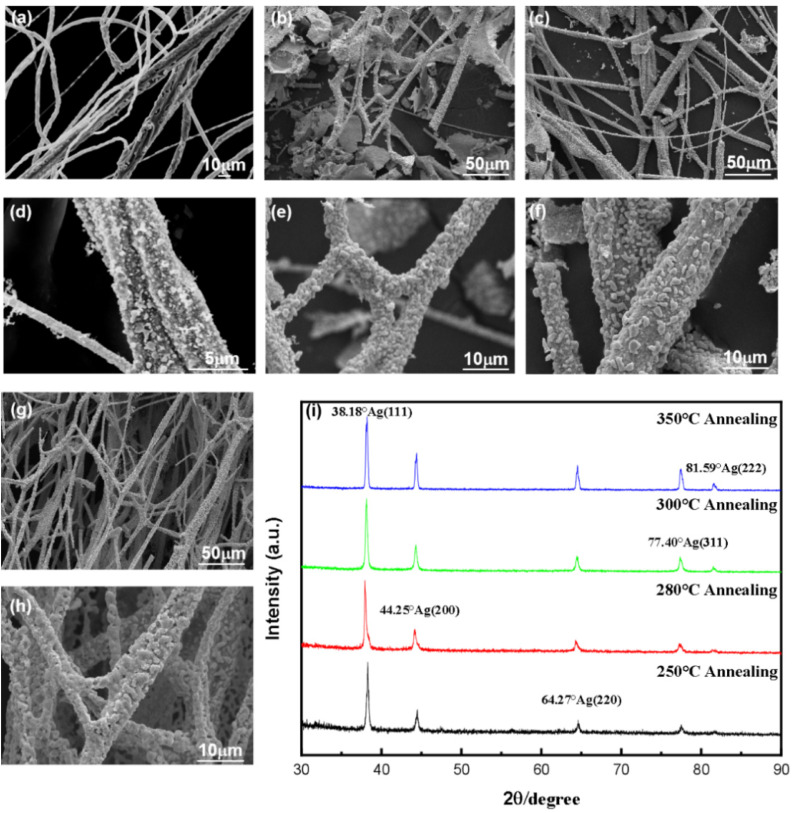
SEM image and characterization of silver microfibers based on the different annealing temperatures. SEM images of silver fibers annealed at 250 °C (**a**,**b**), 280 °C (**c**,**d**), 300 °C (**e**,**f**), and 350 °C (**g**,**h**). (**i**) XRD patterns of centrifugal-spun fibers annealed at different temperatures.

In order to assess the effect of annealing temperature on the formation of ultra-long silver microfibers, a precursor of silver microfibers prepared with PVP:AgNO_3_ = 5:15 and a 30 G needle were annealed at 250, 280, 300, and 350 °C. As shown in Figure 5a–h and Figure S5, the morphology of the silver fibers annealed at different temperatures differed, as a result of the thermal properties of PVP and AgNO_3_. According to the references [17,25], the metal ions, generally in the composite, have two distinct effects on the glass transition temperature (Tg) of polymers. Cross-linking/coordination between Ag cations and polymer electron donor groups reduces chain mobility and increases Tg. The distributed complexes in the polymer, on the other hand, reduce the crystallinity and Tg of the polymer because they disrupt the uniformity of the polymer chains. In reference [17], the results show that the addition of Ag ions can disrupt the uniformity of PVP chains since the Tg of the initial PVP polymer decreases from 186.9 to 162.5 °C. For PVP, the second stage was 11.5% less in the temperature range of 250 to 396 °C, and the third stage was 61% less in the temperature range of 396 to 695 °C, indicating that pure PVP began to degrade above 250 °C and completely decomposed at temperatures above 695 °C. It also means that high temperature causes the PVP to decompose rapidly, releasing gas and affecting the morphology of the fiber, causing fiber fracture, as shown in Figure 5a–h. In contrast, as the temperature rises, the size of the silver particles on the surface increases, and the connections between the silver particles form a new shell for the PVP/AgNO_3_ fiber. Further more, higher Mw of PVP results in better thermal properties of PVP and a stronger fiber that resists centrifugal force and is less likely to break during the spinning process as shown in Figure S6.

The characteristic peaks of silver (111, 200, 220, 311, and 222) were observed in the annealed PVP/AgNO_3_ fiber XRD patterns, as shown in Figure 5i. The crystalline size of the silver particle was calculated. When the annealing temperature was raised from 250 to 300 °C, the size of the silver particle increased from 25.2 to 65.9 nm. However, when the temperature was raised to 350 °C, the size of the silver particle decreased to 55 nm. Essentially, the annealing procedure at high temperatures promotes Ag crystal growth while shortening fiber lengths and destroying morphology.

### 3.5. Electrical Properties of the Silver Microfiber

As illustrated in Figure S7, the pristine PVP/AgNO_3_ film (weight ratio = 5:15) on the glass substrate does not have conductive properties because it lacks a conducting network constructed with conductive elements, which correspond to the Ag^+^ that has not been converted into silver. The film exhibits a sheet resistivity of 500 Ω/□ after 5 min of annealing at 50 °C, illustrating the low conductive properties of the film. In other words, because some Ag^+^ has been converted into silver, the annealed film possesses a conductive network. The annealing temperature and time were increased in order to convert Ag^+^ into silver Ag efficiently, and the sheet resistivity of the film was as low as 567 Ω/□ after being annealed at 260 °C for 30 min, which was 10 times lower than the film that was annealed at 250 °C for 30 min. This means that increasing the annealing temperature results in reduction of Ag^+^ to Ag, which yields a better conductive network. The results were consistent with the XRD patterns of centrifugally spun fibers after annealing, as illustrated in Figure 5.

**Figure 6:**
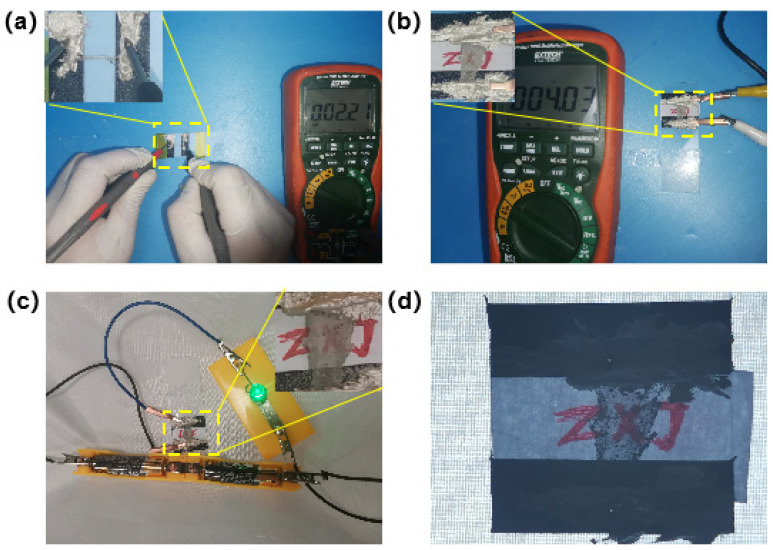
The resistance of a silver fiber bundle (**a**) and a silver-fiber network (**b**). (**c**) The highly conductive silver-fiber network illuminates a bulb at 3 V. (**d**) Demonstrated transparency of the highly conductive silver-fiber network.

The electrical properties of silver fibers prepared with centrifugal spinning were also investigated, as shown in Figure 6 and Figure S8. A silver fiber bundle shows a resistance of 2.2 Ω at a length of 1 cm, while the silver fiber network shows a resistance of 4 Ω at a size of 1 × 0.5 cm, illustrating the high conductivity of the silver fiber prepared by centrifugal spinning paired with annealing (Figure 6a,b). It was also confirmed by a bulb being lit by a 3-Volt battery when connected to a silver-fiber network in a circuit. Furthermore, the letter “X” between Z and J can still be seen after being covered by the silver-fiber network, demonstrating a certain transparency.

## 4. Discussion and Outlook

In this study, centrifugal spinning has been introduced to produce silver microfibers from PVP/AgNO_3_ resources. The process still faces several obstacles on a larger scale, including difficulties in achieving fiber diameter homogeneity, difficulty creating silver fiber with diameters smaller than 100 nm, and difficulty preparing silver fibers with a smooth surface.

**Figure 7:**
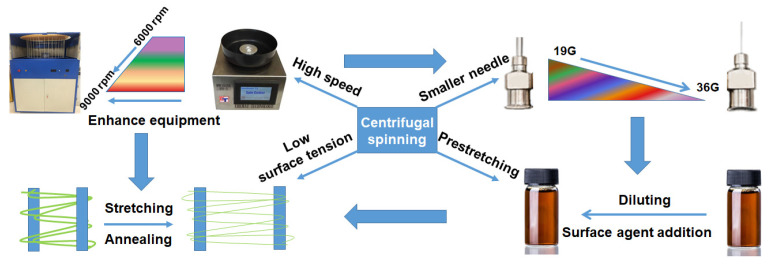
Schematic illustration of a method to reduce the diameter of centrifugal-spun fiber.

As the most important parameter, the diameter of the fiber has significant impact on its properties. Generally, the smaller the diameter of the fiber, the higher the specific surface area and unique properties of the fiber, such as surface effect, small size effect, and quantum size effect. As a result, being able to control the diameter of the silver fiber prepared with centrifuge spinning is valuable and required. Fortunately, it is possible to realize this using the following method, as shown in Figure 7. First, use a smaller needle. The size of the needle determines the initial size of the extruded precursor fiber, directly affecting the diameter of the annealed fiber. Second, reduce the viscosity of the spinning solution. This is necessary because a solution with a lower viscosity can be more easily extruded and elongated by centrifugal force, resulting in smaller diameter fibers. The third modification is the adoption of higher spin speeds. Higher speed results in higher centrifugal force. Of course, the second stretch can reduce the diameter of the fiber before annealing. For example, most polymers relax after heating due to the increased slip ability of chain segments. It means that we can stretch the precursor of silver fiber at a temperature lower than the annealing temperature in order to reduce the diameter of the silver fiber. The smoothness of the silver fiber can be achieved by adjusting both the annealing temperature and time, since they directly affect the crystal size of the silver nanoparticles. Alternatively, different reductants can be used to adjust the rate of silver ion reduction, controlling its growth rate to become more regular.

In order to meet the increasing need for nanofibers with different functions, it is necessary to increase both yield and -length. This could be addressed by two independent but related techniques based on the high yields from centrifugal spinning. For ultra-long silver fibers, as an example, these could be achieved by preparing fiber with different functional materials, such as precursors of Au, Cu, and TiO_2_ nanowires. After post-treatment, the fiber precursor could be transformed into a fiber with a different specific function and morphology. Alternatively, due to the different structures on the surface of the material which provide different properties to them, modifying the surface of ultra-long fiber prepared with centrifugal spinning would be an effective method to give the fiber various nano effects, such as surface effect, small size effect, and quantum size effect. Meanwhile, different fiber structures could be realized by growing nanoparticles or nanowires on the surface of the fiber, and through corrosion of the fiber result in different shapes. Above all, the centrifugal spinning process could be improved by enhancing machines and through experimental design. The method would be an effective way to prepare functional nanofiber with high yields and ultra-long lengths, which can be further functionalized with nanostructures through surface engineering.

## 5. Conclusions

Centrifuge spinning combined with annealing in addition to the help of PVP resulted in the successful preparation of ultra-long silver microfibers. The minimum diameter of the silver fiber obtained was 310 nm as a result of the spinning machine and the size of the needle used. The results showed that a high PVP loading is beneficial to spinning and prevents silver oxidation. The excess PVP reduces the size of the crystal of the Ag nanoparticle. The diameter of the silver fiber decreases as the needle size decreases. Furthermore, a higher annealing temperature benefits silver crystal growth but results in fiber breakage, destroying the morphology of the fiber and shortening the fiber. The strategies for preparation of ultra-long nanofiber were proposed based on centrifugal spinning. Used as a novel method for preparing functional micro/nanofiber, the process could serve as a model for preparing other functional micro/nanofibers, such as CuNWs, AuNWs, and other specific functional materials in nanofiber; moreover, the method could be further functionalized by surface engineering.

## Data Availability

All data supporting the conclusions of this article are included within this article.

## Supplementary Materials

The following supporting information can be downloaded at: https://www.mdpi.com/article/10.3390/nano12132145/s1, Figure S1. SEM image of the precursor of silver fiber prepared by centrifuge spinning under the condition of PVP:AgNO_3_ = 4:15 before (a) and after (b) annealing at 250 °C through 30 G needle. SEM image of the precursor of silver fiber prepared by centrifuge spinning under the condition of PVP:AgNO_3_ = 5:15 before (c) and after (d) annealing at 250 °C through 30 G needle. SEM image of the precursor of silver fiber prepared by centrifuge spinning under the condition of PVP:AgNO_3_ = 6:15 before (e) and after (f) annealing at 250 °C through 30 G needle. Figure S2. SEM image of the precursor of silver fiber and its magnification prepared by centrifuge spinning under the condition of PVP:AgNO_3_ = 5:15 and 250 °C annealing through 30 G needle (a,b), 30 G needle (c,d), and 30 G needle (e,f). Figure S3. The original SEM image (a) of the EDS image (b) relates to the Ag element. Figure S4. SEM image of annealed silver fiber and its magnification prepared by centrifuge spinning under the condition of PVP:AgNO_3_ = 5:15 and 250 °C annealing through 30 G needle (a,b), 30 G needle (c,d) and 30 G needle (e,f). Figure S5. SEM image of annealed silver fiber and its magnification prepared by centrifuge spinning under the condition of PVP:AgNO_3_ = 5:15 and 30 G needle through 30 G needle (a,b), 30 G needle (c,d), and 30 G needle (e,f). Figure S6. SEM image of fiber precursor prepared prepared by PVP with Mw = 1300 K (a–c)and Mw = 360 K (d–f). Figure S7. Electrical properties of PVP/AgNO_3_ solutions (weight ratio: 5:15) spun coating on the glass substrate annealed at different temperatures and times. Figure S8. A single silver fiber showing resistance of 2.15 kΩ (a) and the corresponding SEM image presenting nanostructured fiber surface (b).
