# Supplementary material for: Centrifugal Spinning Enables the Formation of Silver Microfibers with Nanostructures
Source: Nanomaterials (Basel). 2022 Jun 22;12(13):2145. doi: 10.3390/nano12132145 (PMC9268077; doi:10.3390/nano12132145)
Supplement: Supplementary file 1 [file nanomaterials-12-02145-s001.zip › nanomaterials-1755681-Supplementary.pdf]

nanomaterials

Supplementary Materials

Centrifugal Spinning Enables the Formation of Silver Microfibers with Nanostructures

Xujing Zhang [1], Songsong Tang [1,2], Zhaokun Wu [1], Ye Chen [3], Zhen Li [4], Zongqian Wang [2,*] and Jian Zhou [1,*]

[1] Key Laboratory for Polymeric Composite & Functional Materials of Ministry of Education, Guangzhou Key Laboratory of Flexible Electronic Materials and Wearable Devices, State Key Laboratory for Optoelectronic Materials and Technologies, School of Material Science and Engineering, Sun Yat-sen University, Guangzhou 510275, China; xzhangxj@163.com (X.Z.); songsongtang@outlook.com (S.T.); yzwzk613@gmail.com (Z.W.)

[2] School of Textiles and Garment, Anhui Polytechnic University, Wuhu 241000, China

[3] State Key Laboratory for Modification of Chemical Fibers and Polymer Materials, Donghua University, Shanghai 200051, China; chenye@dhu.edu.cn (Y.C.)

[4] Foshan City Zhongrou Material Technology Co., Ltd., Foshan 528225, China; lizhenzuokeyan@163.com (Z.L.)

* Correspondence: wzqkeyan@126.com (Z.W.); zhouj296@mail.sysu.edu.cn (J.Z.)

Citation: Zhang, X.; Tang, S.; Wu, Z.; Chen, Y.; Li, Z.; Wang, Z.; Zhou, J. Centrifugal Spinning Enables the Formation of Silver Microfibers with Nanostructures. *Nanomaterials* **2022**, *12*, 2145. https://doi.org/10.3390/nano12132145

Academic Editors: Takuya Kitaoka and Saverio Mannino

Received: 19 May 2022
Accepted: 20 June 2022
Published: 22 June 2022

Publisher's Note: MDPI stays neutral with regard to jurisdictional claims in published maps and institutional affiliations.

Copyright: © 2022 by the authors. Submitted for possible open access publication under the terms and conditions of the Creative Commons Attribution (CC BY) license (http://creativecommons.org/licenses/by/4.0/).

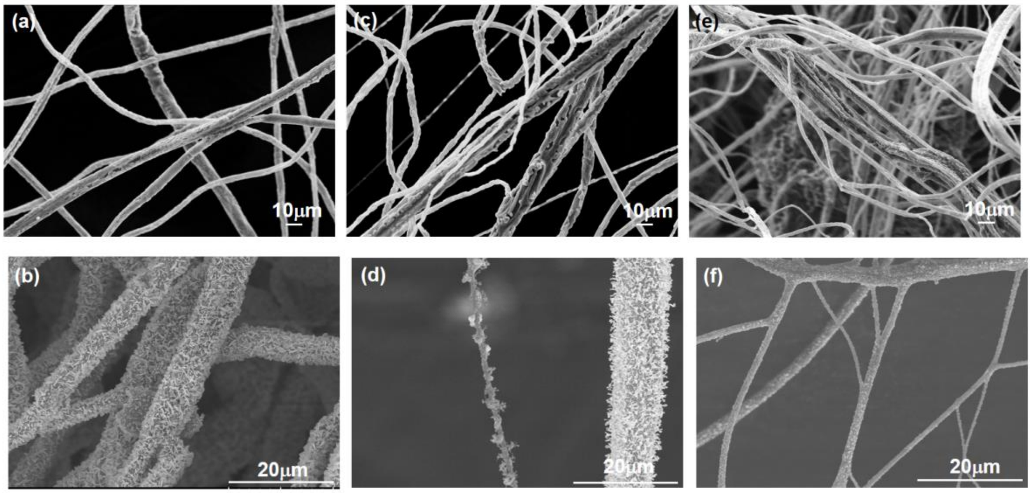

Figure S1. SEM image of the precursor of silver fiber prepared by centrifuge spinning under the condition of PVP: $AgNO_3$=4:15 before (a) and after (b) annealing at 250°C through 30G needle. SEM image of the precursor of silver fiber prepared by centrifuge spinning under the condition of PVP: $AgNO_3$=5:15 before (c) and after (d) annealing at 250°C through 30G needle. SEM image of the precursor of silver fiber prepared by centrifuge spinning under the condition of PVP: $AgNO_3$=6:15 before (e) and after (f) annealing at 250°C through 30G needle.

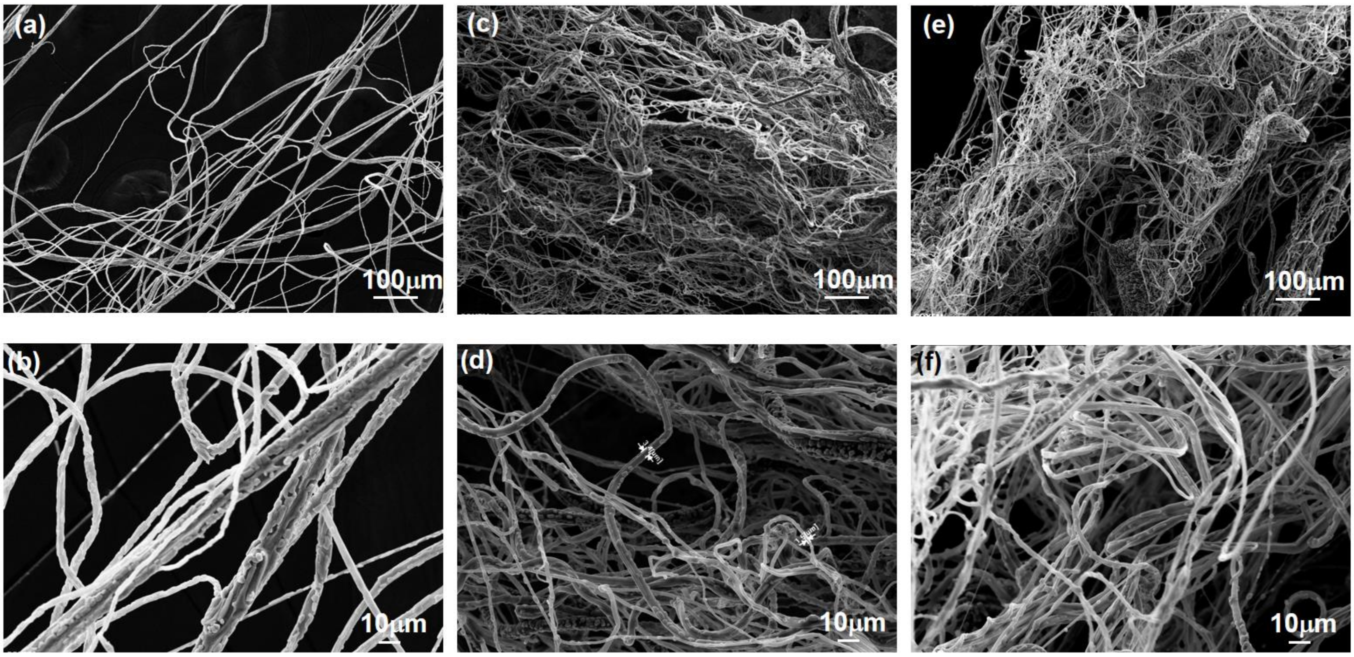

Figure S2. SEM image of the precursor of silver fiber and its magnification prepared by centrifuge spinning under the condition of PVP: $AgNO_3$=5:15 and 250 °C annealing through 30G needle (a and b), 32G needle (c and d), and 34G needle (e and f).

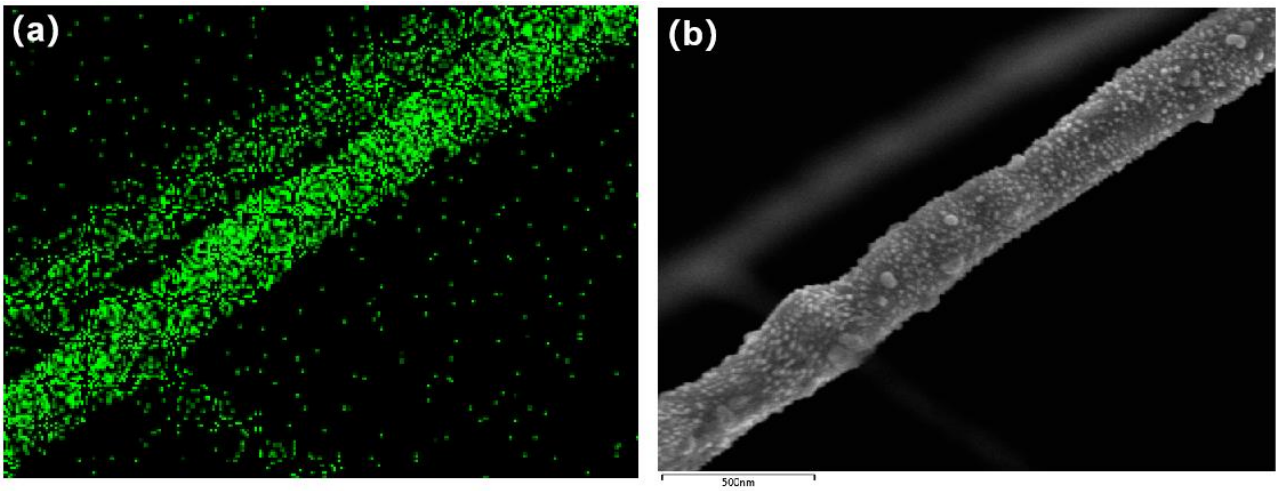

Figure S3. The original SEM image (a) of the EDS image (b) relates to the Ag element.

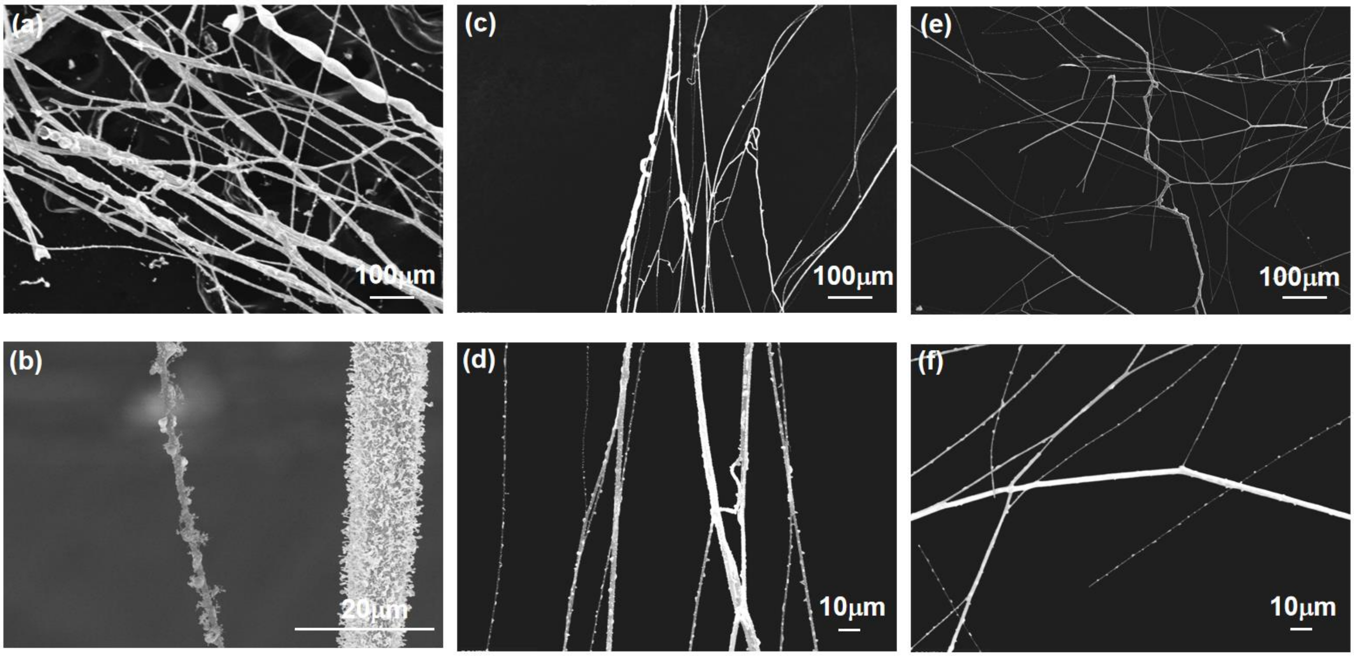

Figure S4. SEM image of annealed silver fiber and its magnification prepared by centrifuge spinning under the condition of PVP: $AgNO_3$=5:15 and 250 °C annealing through 30G needle (a and b), 32G needle (c and d) and 34G needle (e and f).

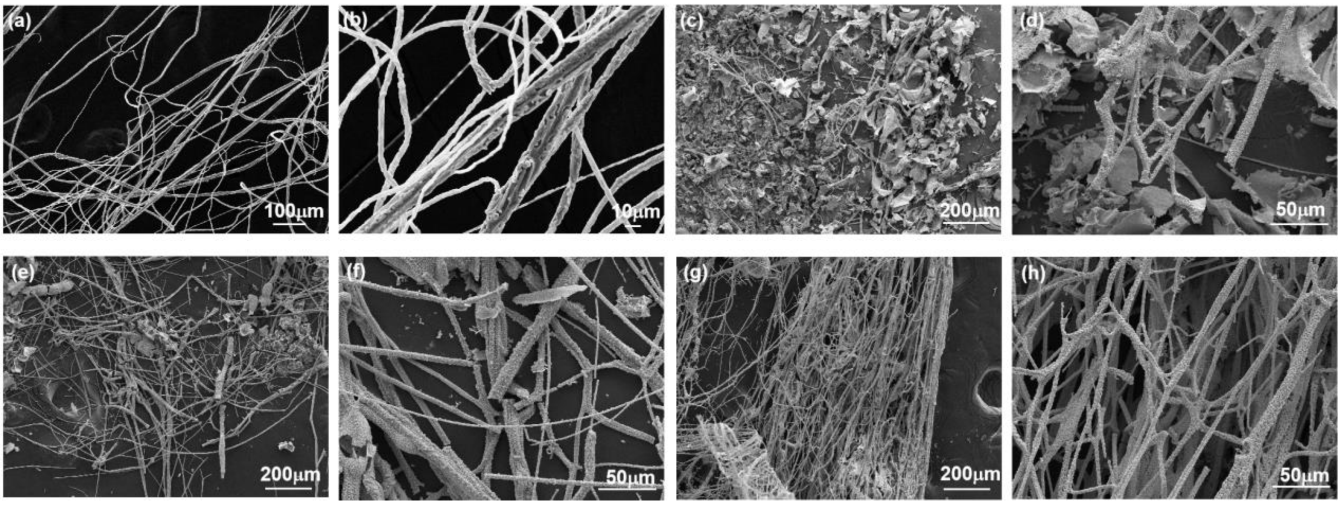

Figure S5. SEM image of annealed silver fiber and its magnification prepared by centrifuge spinning under the condition of PVP: $AgNO_3$=5:15 and 30G needle through 250 °C (a and b), 280 °C (c and d), and 300 °C (e and f). and 350 °C (g and h)

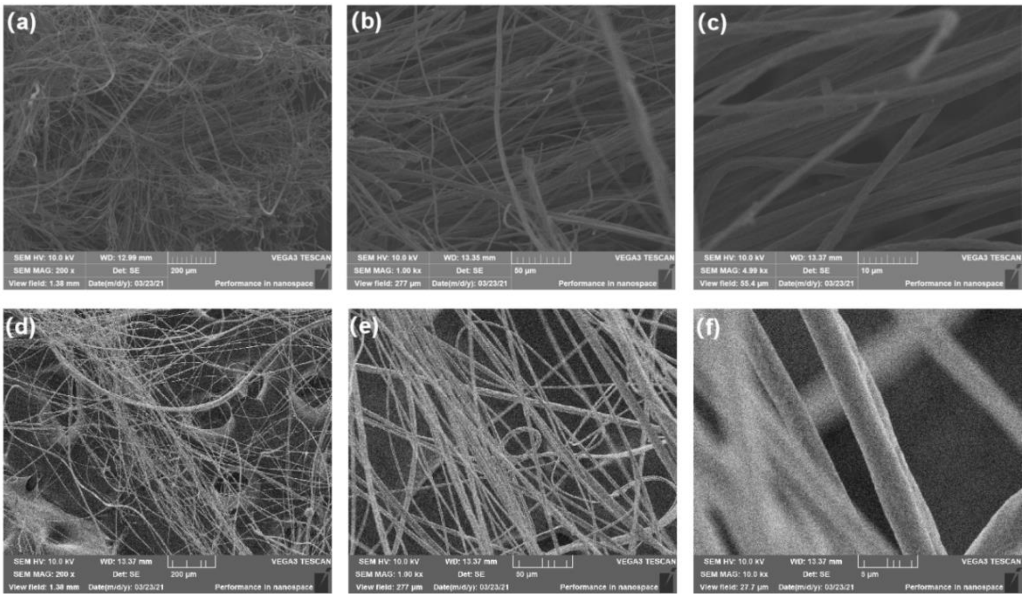

Figure S6. SEM image of fiber precursor prepared prepared by PVP with Mw=1300K (a, b and c)and Mw=360K (d, e and f).

We chose PVP with a Mw of 1300K to prepare longer fibers because the higher Mw of PVP results in a stronger fiber that resists centrifugal force and is less likely to break during the spinning process. Indeed, we compared fibers prepared by PVP with Mw=1300K and Mw=360K, with SEM images shown in Figure S5. The fiber prepared with PVP at Mw=1300K has a more evenly distributed diameter and fewer defects, whereas the fiber prepared with PVP at Mw=360K has a smaller diameter.

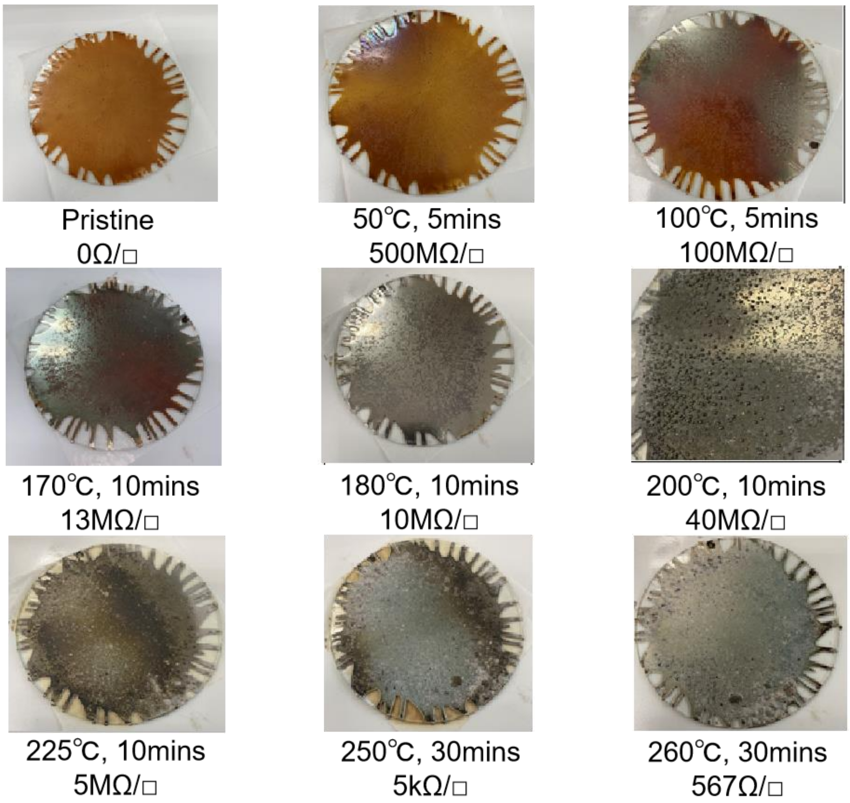

Figure S7. Electrical properties of PVP/$AgNO_3$ solutions (weight ratio: 5:15) spun coating on the glass substrate annealed at different temperatures and times.

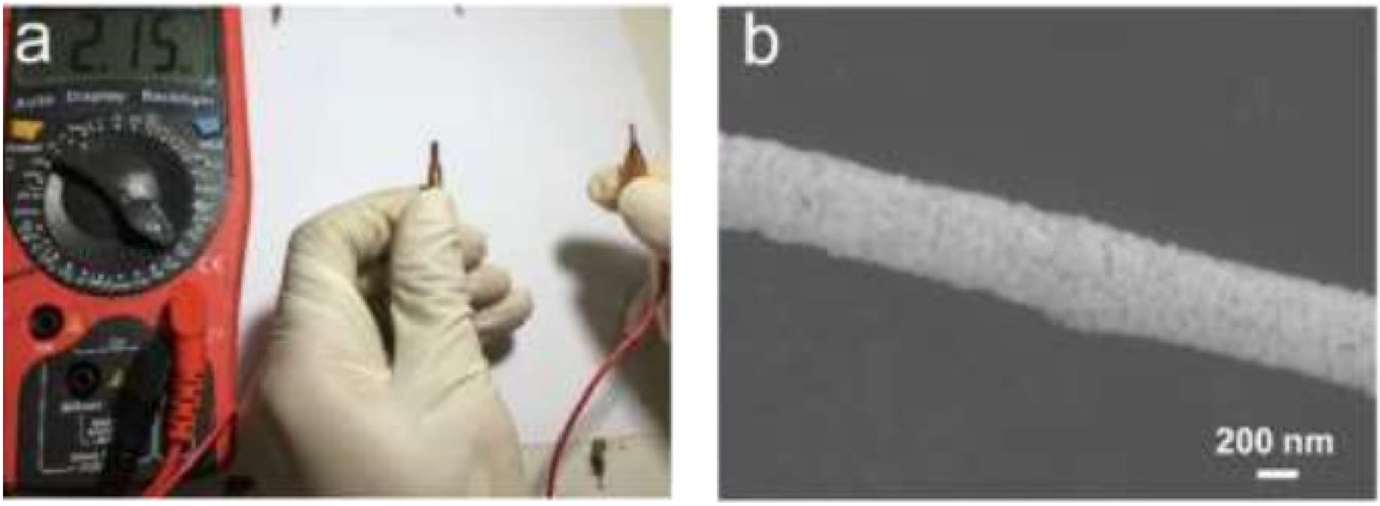

Figure S8. A single silver fiber showing resistance of 2.15 kΩ (a) and the corresponding SEM image presenting nanostructured fiber surface (b).
